# Transcriptomes of developing fruit of cultivated and wild tomato species

**DOI:** 10.1186/s43897-023-00060-5

**Published:** 2023-06-26

**Authors:** Adi Doron-Faigenboim, Michal Moy-Komemi, Marina Petreikov, Yelena Eselson, Prashant Sonawane, Pablo Cardenas, Zhangjun Fei, Asaph Aharoni, Arthur A. Schaffer

**Affiliations:** 1 Plant Sciences Institute, Agricultural Research Organization-Volcani Center, Rishon LeZion, Israel; 2 Department of Plant and Environmental Sciences, Weizmann Institute of Science, Rehovot, Israel; 3 Boyce Thompson Institute, Ithaca, NY USA

Tomato (*Solanum lycopersicum*) is one of the world’s most extensively cultivated crops, and has been the subject of hundreds of years of breeding and selection. Nevertheless, the genetic variability available for the breeding and improvement of tomato within the confines of the species is limited. This has been described as a “genetic bottleneck” (Miller and Tanksley 1990) and is due to the domestication history of the crop, particularly the transfer of select germplasm from South America to Europe in the 1500 s, followed by selections and return to the New World, again of limited germplasm (Knapp and Peralta 2016).

Reaching beyond the *S. lycopersicum* species as a source for genetic variability began nearly 100 years ago, with the introduction of *Cladosporium* resistance from *S. pimpinellifolium* in 1934. As might be expected, the wild species have contributed to breeding for resistances in the cultivated tomato. Surprisingly, and counterintuitively, wild species can contribute to the breeding for improved quality of the fruit (e.g., Rick 1974; Schaffer et al. 1999; Tiemann et al. 2017; Zhao et al. 2019; Pereira et al. 2021) even though the wild species fruit are not of high quality and some of the more primitive wild species are inedible and poisonous.

The potential of wild species to contribute quality traits valuable to tomato improvement is great, but only partially explored and utilized, even since the earlier realization of this potential (Rick 1974; Zamir 2001). Partial metabolomic characterizations of fruit of select wild species and their respective introgression lines indicate the potential inherent in wild species germplasm for modifying primary and secondary metabolite levels in tomato fruit.

The genetic variability for a particular trait can mainly be attributed to two main features of the gene determining the trait: the developmental expression levels of the particular gene, and its sequence polymorphism, which may lead to functionally significant sequence differences, either at the nucleotide or amino acid level. Whole transcript RNA-seq transcriptome analysis offers the advantage of providing both expression and coding sequence polymorphism information, and both measures of genetic variability can be valuable in identifying potential wild species donors for selected genetic traits.

In this paper we report and make available to the research community an extensive data of gene transcript information (whole-transcript RNA-seq) from fruit of 44 tomato accessions, comprising two studies. The first compares transcriptomes of four stages of fruit development, from immature green to ripe, of 16 accessions. These include 4 *lycopersicum*, 2 *pimpinellifolium*, 2 *cheesmaniae*, 3 *chmielewskii*, 2 *habrochaites*, 2 *peruvianum* and a single *pennellii* accession (listed in Supplementary Table S1). The expression data for the developing fruit are presented in Supplementary Table S2. The second study compares the transcriptomes of ripe fruit of 32 additional accessions (listed in Supplementary Table S1), comprising 16 *pimpinellifolium* (8 of Ecuadorian origin and 8 of Peruvian origin), 8 *cheesmaniae* and 8 *galapagense*. These data are presented in Supplementary Table S3. In total, ~ 1.5 billion reads were obtained from 129 libraries derived from 93 samples and mapped against the reference Heinz 1706 genome v4 (Supplementary Table S4).

Irrespective of species group, an initial perusal of the results can give a global overview of gene expression in *Solanum* fruit. Based on the expression results at each of the four developmental stages, approximately 24,000 of the ~ 34,000 annotated tomato genes are fruit-expressed (Fig. 1A). Around 10,000 tomato genes showed no detectable expression or had very low expression (< 10 FPKM) in all the libraries. Most interestingly, expression of ~ 6,000 genes were limited to specific stages of development, with the ripe fruit stage having the most stage-specific expression. Of the 24,123 total fruit-expressed genes the vast majority are expressed in ripe fruit and only 1620 are not expressed in that stage, while 1274 genes are expressed only in the ripe stage.

**Fig. 1:**
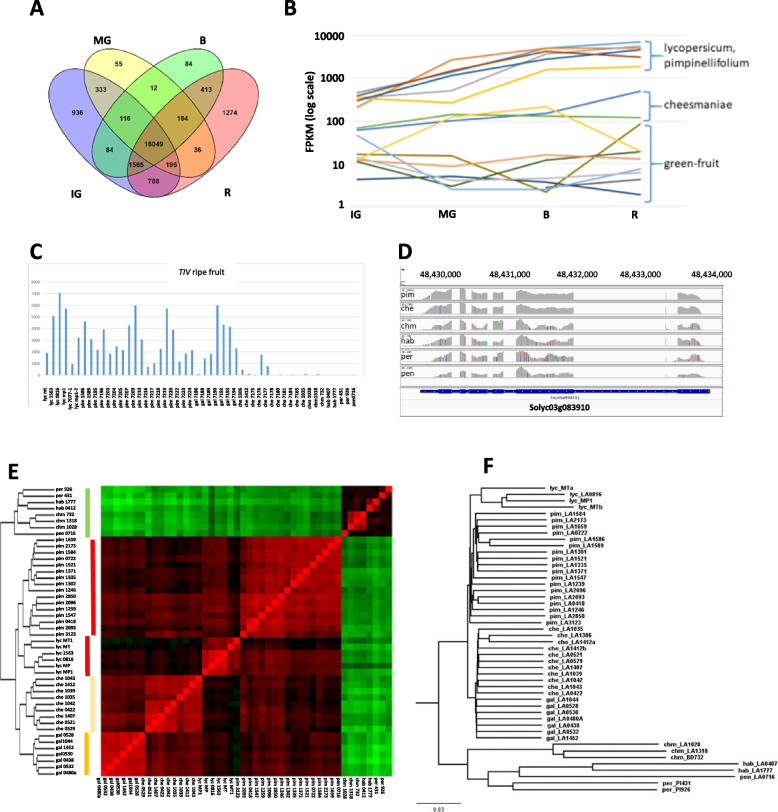
Gene expression among *Solanum* accessions. **A** Venn diagram indicating number of genes expressed in the combined data, arranged according to developmental stages. **B,C** Examples of retrievable data for Solyc03g083910 (*TIV*) for developmental stages (B, Supplementary Table S2) and ripe fruit (C, Supplementary Table S3). **D** IGV screen shot of reads for the six wild species (1 accession each) for Solyc03g083910 (*TIV*). **E,F** Genetic relationships based on **E**) gene expression patterns of ripe fruit and **F**) sequence polymorphisms

The data can be used to screen for natural genetic variation in both gene expression and gene sequences. Figure 1B,C illustrate the results of the two screens for the well-studied soluble vacuolar invertase gene, *TIV*, controlling sucrose/hexose accumulation in the *Solanum* species. Earlier studies (e.g., Schaffer et al. 1999 and references therein) have shown that genetic variation at the *sucr* locus, harboring *TIV,* is responsible for the high concentrations of sucrose in the green-fruited species. Our data are in confluence with these earlier studies that showed that *TIV* expression in green-fruited wild species remains low during ripening, thereby allowing for sucrose accumulation in the fruit, whereas gene expression, and concomitant sucrose hydrolysis, is strongly upregulated in *lycopersicum*, leading to hexose accumulation. The data uncovers additional genetic variability for upregulation, large in *pimpinellifolium* and *galapagense*, but only modest in *cheesmaniae*. In addition, sequence polymorphisms of the TIV alleles can similarly be retrieved (Fig. 1D).

In order to ascertain the significance of transcriptomic patterns to evolutionary and phylogenetic relationships, we compared the phylogenetic tree developed from analysis of transcriptome-derived sequence polymorphisms to the hierarchical tree based on gene expression patterns. SNP calling detected ~ 2.4 M total SNPs, which were filtered to comprise ~ 946 K polymorphic sites identified with a minor allele frequency (MAF) of > 5% across at least 20 accessions (Supplementary file 1). The filtered SNPs were used for calculating distances between each accession to create a neighbor-joining (NJ) tree (Fig. 1F). In comparison, a hierarchical tree and heatmap (Fig. 1E) was generated based on the ripe fruit transcriptomes, utilizing the expression patterns of the ~ 7000 genes that showed at least a fourfold differential expression (adjusted *p* value < 0.001, Supplementary Table S5) between any of the five species groups. The five species groups comprise the accessions of *lycopersicum*, *pimpinellifolium*, *cheesmanaiae*, *galapagense*, and the combined accessions of the primitive green-fruited species, referred to as ‘green species’.

The strikingly similar results between the two approaches strongly indicate that the presumably unbiased evolutionary relationships based on sequence polymorphisms are clearly mirrored by the transcriptional patterns. The green-fruited species are distinctly claded separately from the colored-fruited species, and the colored species exhibit similar relationships between themselves, with both approaches. Both the sequence-based tree and the transcriptome-based relationships point to a common ancestor of the endemic Galapagos species, presumably the founder transferred from the mainland, that itself shared a common ancestor with the green-fruited wild species. Both methods distinguish between the accessions of the two species endemic to the Galapagos Islands, *cheesmaniae* and *galapagense*. Similarly, the two *pimpinellifolium* subgroups, representing Peruvian and Ecuadorian origins (Supplementary Table S1), are distinguished by both methods.

In conclusion, we present a comprehensive data of gene transcripts derived from developing and ripe fruit of cultivated tomato and its wild relatives. The data can serve as a repository for identifying genetic variability in both expression levels and sequence polymorphisms. The latter can identify non-synonymous amino acid sequence differences with its many implications on protein function. The data can also be harnessed for improving the annotated genome, expanding on the *Solanum* pan-genome through a pan-transctriptome and, perhaps most significantly, shedding light on the evolution of the tomato clade and the relationships between the primitive green-fruited wild species, the presumably intermediate stages of tomato evolution (wild, colored-fruited species) and the cultivated tomato.

We have previously utilized this data for the identification of tomato genetic variability and gene identification. These included studies of the plant cholesterol biosynthetic pathway by a multi-species gene co-expression analysis (Sonawane et al. 2016), identification of genes involved in novel glycoalkaloid metabolism (Sonawane et al. 2022), surveys of genetic variability for the SWEET sugar transporter family (Shammai et al. 2018) and for the prenyltransferase family, involved in volatile terpene metabolism (Hivert et al. 2020). Our hope is that this data, combined with other tomato expression databases, such as TED (http://ted.bti.cornell.edu/), TEA (https://tea.solgenomics.net/) and TomExpress (http://tomexpress.toulouse.inra.fr/) will serve the research and breeding communities in furthering the study of tomato genetics and improvement.

### Supplementary Information

**Additional file 1. **

**Additional file 2: Supplemental Table 1.** List of accessions and sources used in this report. **Supplemental Table S2.** Gene expression data (FPKM) for fruit of Solanum accessions at 4 stages of development. Accessions are listed in Supplemental table S1. **Supplemental Table S3.** Gene expression data (FPKM) for ripe fruit of Solanum accessions. Accessions are listed in Supplemental table S1. **Supplemental Table S4.** Mapping statistics for libraries used in this study. **Supplementary Table S5.** Correlation matrix used for generation of Figure 3A, heata map and hierarchical clustering based on differential gene expression.

## Data Availability

The RNA-seq data are available in NCBI BioProject database under the accession numbers PRJNA798612 and PRJNA922439. Expression data will be available at http://ted.bti.cornell.edu/. All other data generated in this study are included in the article and additional files.

## Abbreviations

lyc: *lycopersicum*
che: *cheesmaniae*
gal: *galapagense*
pim: *pimpinellifolium*
chm: *chmieliewskii*
hab: *habrochaites*
per: *peruvianum*
pen: *pennellii*

## References

[CR1] Hivert G, Davidovich-Rikanati R, Bar E, Sitrit Y, Schaffer A, Dudareva N, Lewinsohn E (2020). Prenyltransferases catalyzing geranyldiphosphate formation in tomato fruit. Plant Sci.

[CR2] Knapp S, Peralta IE (2016). The tomato (*Solanum lycopersicum* L, Solanaceae) and its botanical relatives. The tomato genome.

[CR3] Miller JC, Tanksley SD (1990). RFLP analysis of phylogenetic relationships and genetic variation in the genus Lycopersicon. Theor Appl Genet.

[CR4] Pereira L, Sapkota M, Alonge M, Zheng Y, Zhang Y, Razifard H, Taitano NK, Schatz MC, Fernie AR, Wang Y, Fei Z, Caicedo AL, Tieman DM, van der Knaap E (2021). Natural genetic diversity in tomato flavor genes. Front Plant Sci.

[CR5] Rick CM. High soluble-solids content in large-fruited tomato lines derived from a wild green-fruited species. The University of California, Division of Agricultural and Natural Resources; 1974.

[CR6] Schaffer AA, Miron D, Petreikov M, Fogelman M, Spiegelman M, Bnei-Moshe Z, Shen S, Granot D, Hadas R, Dai N, Bar M, Levin I, Friedman M, Pilowsky M, Gilboa N, Chen L (1999). Modification of carbohydrate content in developing tomato fruit. HortScience.

[CR7] Shammai A, Petreikov M, Yeselson Y, Faigenboim A, Moy-Komemi M, Cohen S, Cohen D, Besaulov E, Efrati A, Houminer N, Bar M, Ast T, Schuldiner M, Klemens PAW, Neuhaus E, Baxter CJ, Rickett D, Bonnet J, White R, Giovannoni JJ, Levin I, Schaffer AA (2018). Natural genetic variation for expression of a SWEET transporter among wild species of *Solanum lycopersicum* (tomato) determines the hexose composition of ripening tomato fruit. Plant J.

[CR8] Sonawane PD, Pollier J, Panda S, Szymanski J, Massalha H, Yona M, Unger T, Malitsky S, Arendt P, Pauwels L, Almekias-Siegl E, Rogachev I, Meir S, Cárdenas PD, Masri A, Petrikov M, Schaller H, Schaffer AA, Kamble A, Giri AP (2016). Plant cholesterol biosynthetic pathway overlaps with phytosterol metabolism. Nat Plants.

[CR9] Sonawane PD, Jozwiak A, Barbole R, Panda S, Abebie B, Kazachkova Y, Gharat SA, Ramot O, Unger T, Wizler G, Meir S, Rogachev I, Doron-Faigenboim A, Petreikov M, Schaffer A, Giri AP, Scherf T, Aharoni A (2022). 2-oxoglutarate-dependent dioxygenases drive expansion of steroidal alkaloid structural diversity in the genus *Solanum*. New Phytol.

[CR10] Tieman D, Zhu G, Resende MFR, Lin T, Nguyen C, Bies D, Rambla JL, Beltran KSO, Taylor M, Zhang B, Ikeda H, Liu Z, Fisher J, Zemach I, Monforte A, Zamir D, Granell A, Kirst M, Huang S, Klee H (2017). A chemical genetic roadmap to improved tomato flavor. Science.

[CR11] Zamir D (2001). Improving plant breeding with exotic genetic libraries. Nat Rev Genet.

[CR12] Zhao J, Sauvage C, Zhao J, Bitton F, Bauchet G, Liu D, Huang S, Tieman DM, Klee HJ, Causse M (2019). Meta-analysis of genome-wide association studies provides insights into genetic control of tomato flavor. Nat Commun.

