# Supplementary material for: Transcriptomes of developing fruit of cultivated and wild tomato species
Source: Mol Hortic. 2023 Jun 26;3:12. doi: 10.1186/s43897-023-00060-5 (PMC10514971; doi:10.1186/s43897-023-00060-5)
Supplement: Supplementary file 1 — Additional file 1. [file 43897_2023_60_MOESM1_ESM.docx]

**Supplemental Files**

**Methods and materials**

**Accessions used**

The species used in this study, their description and accession numbers, are listed in Supplementary Table 1. As described in the table, some of the accessions (LAs) were obtained from TGRC (<https://tgrc.ucdavis.edu/>) while others were available at the ARO-Volcani-Bet Dagan tomato seed collection, and are listed as BD numbers. The two *peruvianum* accessions have PI numbers, originally sourced from the USDA-ARS. One of the three *chmielewskii* accessions used in this study is of undetermined provenance and is listed as BD732. Four accessions of *S. lycopersicum*, three of *S. chmielewskii*, two each of *S. pimpinellifolium, S. cheesmaniae, S. habrochaites, S. peruvianum*, and a single accession of *S. pennellii* were sampled through fruit development, as described below. An additional 16 accessions of *S. pimpinellifolium* (8 originating from Peru and 8 from Ecuador), 8 of *S. cheesmaniae* and 8 of *S. galapagense* were grown as above and sampled at the ripe fruit stage only. One of the *lycopersicum* accessions included, LA1563, the "Rick's high sugar line" (Rick, 1974), is derived from a cross with *S*. *chmielewskii* (then *L. minutum*) and harbors a number of small donor introgressions. Nevertheless, it is included among the *lycopersicum* accessions. Two of the *lycopersicum* accessions (MT and MP) and *cheesmaniae* LA1412, grown and sampled for the developmental study, were grown and sampled independently in the experiment comparing ripe fruit of the 32 *pimpinellifolium*, *cheesmaniae* and *galapagense* accessions. Therefore, the combined ripe fruit data (Supplementary Table S3) includes independent duplicate samples of lycMT, lycMP and cheLA1412, as well as the ripe fruit stage of the wild species developmental data (Supplementary Table S2)

**Fruit sampling**

Plants were grown in a greenhouse with supplemental heating to a minimum of 15 ^o^C (developmental study in 2014, ripe fruit study in 2017). Flowers were allowed to self-pollinate or were assisted by sib-pollination without emasculation. A minimum of three plants per accession were grown and developing fruit were sampled at four developmental stages, I-IV. Since the developmental processes of the different wild species are not temporally synchronized, we sampled the developmental stages phenotypically, parallel to the four main developmental stages of *S. lycopersicum*: immature green (IG), mature green (MG), breaker (Br) and ripe (R).

Stage I indicates fruitlets of approximately 2 weeks from anthesis and about half diameter of the full size fruit of the respective accession, analogous to the *lycopersicum* immature green (IG) stage; stage II was chosen from nearly fully developed fruit before any indication of color change, similar to *lycopersicum* mature green (MG). Stage III is the onset of color change, or breaker (Br), which even in the green-fruited species can be discerned as a lightening of the green hue. Stage IV represents the ripe stage (R), which in the case of the green-fruited species was indicated by the beginning of fruit softening. For the non-green-fruited wild species (*S. pimpinellifolium*, *S. galapagense* and *S. cheesmaniae*) which undergo chloroplast to chromoplast conversion and are yellow, orange or red at ripe stage, the stages coincided more clearly with those of *S. lycopersicum*. The additional 32 accessions (16 of *S. pimpinellifolium*, 8 of *S. cheesmaniae* and 8 of *S. galapagense*) were sampled at the ripe fruit stage only. Descriptions of tissues sampled and mapping results of libraries are presented in Supplementary Table 4. Fruits were brought to the laboratory, and seeds were removed before freezing in liq N and stored at -80C until use.

**RNA extraction and RNA-seq**

Samples for each developmental stage from each of the wild species accessions, as well as ripe stage fruit of additional 32 accessions, were bulked and ground to a fine powder in liquid nitrogen. A minimum of 5 fruit comprised each sample. For the *S. lycopersicum* accessions, 3 individual samples of each stage were independently prepared. RNA was extracted using different commercial plant RNA extraction kits, which varied during the period covered in the study. In addition, 12 combined RNA samples, each comprised of the combined RNA of the four developmental stages of the 12 wild species accessions, was prepared and used for a paired end 2 X 150 bp Illumina sequencing. RNA-seq was performed using Illumina technology, which also changed during the period covered in the study. Of the 130 samples, 129 yielded high quality sequencing and mapping results. A single sample, from *S. chmielewskii* LA1028, stage II, is not included in the developmental study due to poor quality results. The library metadata and individual library statistics are presented in Supplementary Table S4.

**Expression analysis**

Raw reads were subjected to a filtering and cleaning procedure. The Trimmomatic tool (Bolger et al., 2014) was used to remove Illumina adapters from the reads. Next, the FASTX Toolkit (<http://hannonlab.cshl.edu/fastx_toolkit/index.html>, version 0.0.13.2) was employed to trim read-end nucleotides with quality scores < 30 using the FASTQ Quality Trimmer, and to remove reads with less than 70% base pairs with a quality score ≤ 30 using the FASTQ Quality Filter. Clean reads were mapped to the reference genome of *Solanum lycopersicum* (<ftp://ftp.solgenomics.net/tomato_genome/assembly/build_4.00/> ) using STAR software (v2.7.1a) (Dobin et al., 2012). Gene abundance was estimated using Cufflinks (v. 2.2) ([Trapnell](https://www.mdpi.com/2073-4409/11/14/2215/htm#B21-cells-11-02215) et al.,2010) combined with gene annotations from the Sol Genomics Network database (<https://solgenomics.net/>; <ftp://ftp.solgenomics.net/tomato_genome/annotation/ITAG4.0_release/> ). Principal component analysis (PCA) and heatmap visualization were performed using R Bioconductor (accessed on 15 May 2022) ([Gentleman](https://www.mdpi.com/2073-4409/11/14/2215/htm#B22-cells-11-02215) et al., 2004). Gene expression values were computed as FPKM. Differential expression analysis was completed using the DESeq2 R package (accessed on 27 April 2021) ([Love](https://www.mdpi.com/2073-4409/11/14/2215/htm#B23-cells-11-02215) et al., 2014). Venn diagrams were performed using the Venn web tool (<https://bioinfogp.cnb.csic.es/tools/venny/index.html>; accessed on 28 April 2021). Hierarchical cluster analysis of heatmaps was performed with the ClustVis tool (accessed on 28 April 2021) ([Metsalu](https://www.mdpi.com/2073-4409/11/14/2215/htm#B25-cells-11-02215) et al., 2015). Cluster analysis of the DEGs based was conducted using Expander 7 software (Ulitzky et al.,2010) with the K-means algorithm (Shamir et al., 200 ). KOBAS 3.0 tool (Xie et al., 2011) (http://kobas.cbi.pku.edu.cn/kobas3/?t=1) was applied to find the statistical enrichment of KEGG pathways and Gene Ontology (GO) terms in differentially expressed genes.

**De-novo transcript assembly**

Clean paired-end reads were assembled de novo using Trinity software (version: v2.1.1; [Grabherr et al. 2011](javascript:;)) with the trimmomatic option to remove adaptors (Grabherr et al., 2011) and k-mer size of 25. The assembled contigs were used as query terms in a BLASTx (Altschul et al., 1990) search against the *Solanum lycopersicum* and the *Solanum pennellii* proteins as well as versus the reference genome sequences (<https://solgenomics.net/>).

**SNP analysis**

The resulting mapping files were processed using Picard tool (http://broadinstitute.github.io/picard/; version 1.95) (Li et al., 2009) for adding read group information, sorting, marking duplicates, and indexing. Then, the local re-alignment process for locally re-aligning reads such that the number of mismatching bases is minimized across all the reads was performed using the Realigner Target Creator of the Genome Analysis Toolkit version  v4.1.9.0  (GATK; <http://www.broadinstitute.org/gatk/>) (DePristo et al., 2011). Finally, the variant calling procedure was performed using HaplotypeCaller of the GATK toolkit, for the detection of SNPs between the variants and the reference. SnpEff tool (version 5.0d) (Cingolan et al., 2012) was used for annotations and predictions the effects of genetic variants based on the annotation downloaded from the Sol Genomics Network database. The phylogenetic tree based on the SNPs was constructed using TASSEL 5 (Bradbury et al., 2007) and the Neighbor-Joining (NJ) method.

**Data availability**

The raw reads have been deposited into NCBI BioProject under accession numbers PRJNA798612 and PRJNA922439. Other data is presented in Supplemental Files and Supplemental Tables.

**Fig. 2. Difference in % reads uniquely mapped to the *lycopersicum* and *pennellii* reference genomes.** Positive and negative values indicate preferential mapping to the *lycopersicum* reference and *pennellii* reference, respectively.

**Fig. 4. Hierarchical clustering based on gene expression of developmental tissues.**

**Fig. 5. Developmental gene expression patterns based on cluster analysis.** A) Cluster analysis for each of the four species groups, *lycopersicum* (lyc), *pimpinellifolium* (pim), *cheesmaniae* (che), combined green-fruited species (green). B) Venn diagrams representing ripening upregulated (left) and downregulated (right) genes according to developmental cluster analysis shown in A. Clusters chosen for each species group are shown and listed in Supplementary Tables S9 and 12.

**Fig. 6. Genes showing differential expression between the green-fruited and combined colored-fruited species at each developmental stage.** A) Number of differentially expressed genes at each stage. B) Venn diagram indicating the overlap of differentially expressed genes among different stages. Differential expression is considered as >2-fold difference and adjusted p <0.05.

**Fig. 7. Genes downregulated in ripe fruit of colored-fruited species.** A) Number of genes downregulated between the combined green-fruited species and each of the colored-fruited species; B) Venn diagram indicating overlap of number of downregulated genes for each colored species; C) KOBAS-based bubble plot indicating enriched GO and KEGG pathways among the 1014 commonly downregulated genes. Only terms which include >50 genes with a P value <0.05 are included in the figure. Data is expressed as –log(P). D) Venn diagram of number of genes silent (<2 FPKM) in each colored-fruited species and expressed >10 FPKM in each green-fruited species. red indicates *lycopersicum* accessions, yellow_che, *cheesmaniae* accessions, yellow_gal, *galapagense* accessions, dark red, *pimpinellifolium* accessions. The complete data is provided in Supplementary File 5.

**Fig. 8. Genes upregulated in ripe fruit of each of the colored fruited species compared to expression in the green fruited species**. A) Number of genes upregulated in each of the colored-fruited species compared to the combined green-fruited species. Genes upregulated >2-fold (P value <0.001) are included in column 2 and those not expressed in the green-fruited species (FPKM <2) and expressed in each of the colored fruited-species at levels >10FPKM are listed in column 3. B) Venn diagram indicating overlap of genes upregulated >2-fold in each colored-fruited species, compared to green-fruited species. C) Venn diagram indicating overlap of genes silenced in green-fruited species. Full data is provided in Supplementary File 5.

**Table 1. Selected novel *S. pennellii* genes not present in the *S. lycopersicum* genome.** Data provided in Supplementary Table S6, based on mapping of *penellii* reads that did not map to the *lycopersicum* genome.

| ***Sopen* gene** | **Closest S*olyc* gene** | **Annotation** | **%identity** |
| --- | --- | --- | --- |
| Sopen10g036130 | Solyc09g011810 | Fructose-1-6-bisphosphatase | 75 |
| Sopen02g036790 | Solyc03g043920 | Galactosyl transferase | 67 |
| Sopen12g014550 | Solyc03g113490 | Glucose dehydrogenase | 62 |
| Sopen03g013100 | Solyc07g065980 | Raffinose synthase | 58 |
| Sopen01g054330 | Solyc04g082700 | Sugar transporter | 70 |
| Sopen02g017320 | Solyc06g082300 | UDP-glucosyl transferase | 55 |

**Table 2. P values of selected KEGG and GO families of the genes shown in Fig. 5.** Highly significant P values are highlighted in red. Common relates to all 4 species groups, colored refers to *lycopersicum*, *pimpinellifolium* and *cheesmaniae*, lyc refers to developmentally regulated only in the *lycopersicum* accessions, and green refers to developmentally regulated only in the primitive green-fruited accessions. Full data are provided in Supplementary tables S10 and S11.

**Table 3. Transcription factors developmentally downregulated and upregulated in *Solanum* species.**

**Table 4: Genes silenced in ripe fruit in *lycopersicum* and significantly expressed (FPKM >10) in ripe fruit of each of the other species.**

**Table 5. Genes silenced in ripe fruit of green-fruited species and expressed in ripe fruits of all colored-fruited species**

| **Gene** | **Annotation** | **green** | **pim** | **lyc** | **che** | **gal** |
| --- | --- | --- | --- | --- | --- | --- |
| Solyc01g097200.1 | Ankyrin-like | 1 | 12 | 16 | 14 | 11 |
| Solyc02g088940.3 | CTP synthase | 2 | 46 | 44 | 45 | 69 |
| Solyc01g098530.3 | CTP synthase | 2 | 42 | 26 | 35 | 24 |
| Solyc05g011970.3 | Cytochrome | 2 | 27 | 41 | 38 | 23 |
| Solyc02g082633.1 | DNA mismatch repair | 0 | 23 | 22 | 41 | 58 |
| Solyc06g063060.3 | Dormancy/auxin associated | 1 | 22 | 13 | 46 | 25 |
| Solyc01g106110.2 | F-box protein | 0 | 10 | 12 | 15 | 15 |
| Solyc11g006120.1 | Glycosyltransferase | 2 | 16 | 27 | 21 | 21 |
| Solyc11g013380.1 | GrpE protein | 0 | 16 | 22 | 18 | 24 |
| Solyc03g059140.4 | Ion channel CASTOR | 1 | 13 | 27 | 19 | 19 |
| Solyc10g074730.3 | Late embryogenesis abundant | 1 | 18 | 16 | 132 | 60 |
| Solyc04g009453.1 | Mediator of RNA pol II | 1 | 10 | 10 | 10 | 12 |
| Solyc04g009480.1 | Mediator of RNA pol II | 1 | 20 | 17 | 21 | 16 |
| Solyc09g018830.2 | MLO-like protein | 1 | 28 | 13 | 23 | 71 |
| Solyc01g098520.3 | MUTL protein | 1 | 12 | 29 | 19 | 20 |
| Solyc02g082770.3 | Phosphoinositide phosphatase | 1 | 20 | 18 | 31 | 51 |
| Solyc11g008100.3 | Translocase subunit seca | 1 | 11 | 11 | 14 | 14 |
| Solyc02g089510.3 | Unknown protein | 0 | 20 | 36 | 13 | 44 |
| Solyc02g089490.4 | Unknown protein | 2 | 23 | 42 | 20 | 32 |
| Solyc02g089530.3 | Unknown protein | 0 | 14 | 19 | 12 | 39 |
| Solyc11g062030.1 | Unknown protein | 0 | 105 | 110 | 181 | 281 |
| Solyc11g068700.2 | Unknown protein | 0 | 205 | 55 | 531 | 218 |
| Solyc12g019700.1 | Unknown protein | 1 | 176 | 275 | 223 | 162 |
| Solyc01g087000.3 | Unknown protein | 2 | 19 | 11 | 11 | 11 |
| Solyc06g011575.1 | Unknown protein | 1 | 36 | 75 | 84 | 62 |
| Solyc03g115985.1 | Unknown protein | 1 | 53 | 76 | 27 | 57 |
| Solyc03g121825.1 | Unknown protein | 0 | 17 | 26 | 13 | 16 |
| Solyc12g089145.1 | Unknown protein | 0 | 20 | 22 | 16 | 17 |
| Solyc07g063465.1 | Unknown protein | 0 | 18 | 16 | 18 | 12 |
| Solyc02g037590.3 | Unknown protein | 0 | 31 | 16 | 37 | 80 |
| Solyc11g032190.1 | Unknown protein | 0 | 52 | 113 | 114 | 162 |
| Solyc03g083640.1 | Unknown protein | 0 | 15 | 27 | 54 | 34 |
| Solyc02g091850.1 | Unknown protein | 0 | 58 | 72 | 124 | 97 |
| Solyc01g150168.1 | Unknown protein | 0 | 229 | 417 | 192 | 267 |
| Solyc06g054220.2 | Unknown protein | 1 | 24 | 26 | 42 | 52 |
| Solyc11g065420.3 | Unknown protein | 0 | 18 | 38 | 76 | 79 |
| Solyc09g059650.4 | Unknown protein | 0 | 25 | 50 | 53 | 83 |

**Table 6. Genes silent in all wild species and expressed in ripe fruit of only *lycopersicum*.**

| **Gene** | **Annotation** | **green** | **pim** | **lyc** | **che** | **gal** |
| --- | --- | --- | --- | --- | --- | --- |
| Solyc08g021870.3 | Ankyrin repeat | 0 | 1 | 13 | 0 | 0 |
| Solyc08g066705.1 | Gag-pol polyprotein | 0 | 0 | 22 | 0 | 0 |
| Solyc09g056180.3 | Glutathione S-transferase | 0 | 0 | 13 | 0 | 0 |
| Solyc12g006980.2 | Leucine-rich repeat | 1 | 0 | 20 | 0 | 1 |
| Solyc08g150134.1 | Ser/thr-protein phosphatase | 0 | 1 | 12 | 0 | 0 |
| Solyc03g096220.3 | tRNA-splicing endonuclease | 1 | 0 | 13 | 0 | 0 |
| Solyc01g008400.3 | Unknown protein | 1 | 1 | 18 | 1 | 0 |
| Solyc03g095880.1 | Unknown protein | 0 | 1 | 311 | 0 | 0 |
| Solyc04g053040.3 | Unknown protein | 0 | 0 | 12 | 0 | 0 |
| Solyc07g056685.1 | Unknown protein | 1 | 0 | 34 | 0 | 0 |
| Solyc10g024365.1 | Unknown protein | 0 | 2 | 15 | 0 | 0 |

**Table 7. List of genes with sequence polymorphisms leading to stop codons in one or more of the species groups.** The *cheesmaniae* and *galapagense* sequences share the polymorphisms and are combined. Stop codons are represented by *.

**Supplementary Information**

The online version contains supplementary information available at <https://doi>.

Supplementary Files will be deposited at figshare (<https://figshare.com/>).

**Supplementary File 1.** De novo assemblies of wild species accessions.

**Supplementary File 2.** VCF file of polymorphisms derived from transcriptome reads.

**Supplementary File 3.** KEGG and GO analyses of clusters.

**Supplementary File 4.** Differentially expressed genes at individual developmental stages.

**Supplementary File 5.** Lists of differentially expressed genes from ripe fruit.

**Supplementary Fig. S1.** Hierarchical clustering of individual developmental stages.

**Supplementary Fig. S2.** Developmental expression ofSolyc01g079360 WRKY.

**Supplementary Fig. S3.** Screen shot of IGV of Solyc08g005160.

**Supplementary Fig. S4.** Screen shot of IGV of region Solyc01g015166 to Solyc01g015168.

**Supplementary Fig. S5.** IGV screenshot indicating reads in the region of Solyc03g095880.

**Supplementary Table S1.** Description of accessions used in this study.

**Supplementary Table S2.** Expression data (FPKM) of developing fruit of *Solanum* accessions.

**Supplementary Table S3.** Expression data (FPKM) of ripe fruit of *Solanum* accessions

**Supplementary Table S4.** Library and mapping statistics.

**Supplementary Table S5**. Results of remapping unmapped *pennellii* reads to *pennellii* reference genome.

**Supplementary Table S6.** Novel *pennellii* genes not present in *lycopersicum* genome.

**Supplementary Table S7.** List of *pennellii* genes present in *lycopersicum* genome but not annotated in ITAG 4.0.

**Supplementary Table S8.** Expression data of ripe fruit used for Figure 3A.

**Supplementary Table** **S9.** List of genes from developmental cluster analysis (Figure 5)

**Supplementary Table S10.** List of genes developmentally upregulated in all species.

**Supplementary Table S11.** List of genes developmentally downregulated in all species.

**Supplementary Table S12.** List of transcription factors downregulated in ripe colored-fruited accessions.

**Supplementary Table S13.** Developmental expression of genes silenced in ripe *lycopersicum*.

**Supplementary Table S14**. Developmental expression of genes upregulated in ripe *lycopersicum*.

**Supplementary Table S15**. List of snps distinguishing between accessions of this study with effect on translation.

**Supplementary Table S16.** List of genes with polymorphism leading to stop codon.
